# Requirements and attitudes toward eHealth content in human medicine studies: A cross-sectional study to improve the curriculum

**DOI:** 10.3205/000355

**Published:** 2026-01-13

**Authors:** Pia Traulsen, Tjorven Stamer, Jost Steinhäuser

**Affiliations:** 1 Institut für Allgemeinmedizin, Universitätsklinikum Schleswig-Holstein, Campus Lübeck, Germany

**Keywords:** eHealth, telemedicine, undergraduate training, students, alumni, curriculum, Germany

## Abstract

**Objective:**

This study investigates the current integration of eHealth content into the medical curriculum at the University of Luebeck. The aim is to explore how students and alumni remember e-health teaching content und to assess their knowledge, confidence and preparedness for using digital applications in clinical practice. The results can help with the further development of the curriculum.

**Methods:**

A cross-sectional online survey was conducted among current medical students and alumni (graduation within the last five years) of the University of Luebeck. The questionnaire assessed participants’ attitudes toward eHealth, their self-assessed knowledge and confidence, and the eHealth content taught during their studies. Data were analyzed descriptively, and associations between key variables were examined using Spearman’s rank correlation and Mann-Whitney U test.

**Results:**

A total of 131 responses were analyzed (students: n=76, alumni: n=54). While students reported more frequent exposure to eHealth content (70%) than alumni (19%), overall, 51% indicated that no eHealth instruction had been part of their curriculum. The most common taught topics included the electronic patient record (ePA), ePrescriptions, and communication platforms like Communication in healthcare (KIM) and Telematics messenger (TIM).

Self-assessed knowledge of eHealth showed strong correlations with perceived confidence and preparedness. Confidence in using eHealth applications strongly predicted perceived preparedness. Gender and age were also found to be relevant factors: male participants reported higher knowledge levels, and younger participants more frequently encountered eHealth content.

**Conclusions:**

The findings highlight substantial variability in the perception of eHealth teaching and reveal significant associations between perceived knowledge, confidence, and readiness for professional application. Participants expressed a clear need for broader and more structured digital health education. Core topics identified for future curricular integration include digital documentation systems, telemedicine, data protection, and the use of AI in healthcare. Comprehensive, longitudinal integration of eHealth across core and elective curricula is essential to adequately prepare future physicians for an increasingly digitalized healthcare system.

## Zusammenfassung

**Zielsetzung:**

Diese Studie untersucht die aktuelle Integration von eHealth-Inhalten in das Medizinstudium an der Universität zu Luebeck. Ziel ist es zu erheben, wie Studierende und Alumni die Lehre zu eHealth-Aspekten erinnern und ihr Wissen, ihre Sicherheit und ihre Vorbereitung auf den Einsatz digitaler Anwendungen in der klinischen Praxis einschätzen. Die Ergebnisse können bei der Weiterentwicklung des Curriculums helfen.

**Methoden:**

Es wurde eine Querschnittsstudie mittels Online-Fragebogen unter derzeit eingeschriebenen Medizinstudierenden sowie Absolvent*innen (Abschluss innerhalb der letzten fünf Jahre) der Universität zu Luebeck durchgeführt. Der Fragebogen erfasste Einstellungen gegenüber eHealth, das subjektiv eingeschätzte Wissen, die Nutzungskompetenz sowie die im Studium vermittelten Inhalte. Die Daten wurden deskriptiv ausgewertet, Zusammenhänge mittels Spearman-Rho-Korrelationen und Mann-Whitney-U-Test untersucht.

**Ergebnisse:**

Insgesamt wurden 131 Fragebögen ausgewertet (Studierende: n=76, Alumni: n=54). Während 70% der Studierenden angaben, eHealth-Inhalte im Studium behandelt zu haben, traf dies nur auf 19% der Alumni zu. Insgesamt berichteten jedoch 51% der Befragten, dass eHealth kein Bestandteil ihres Curriculums war. Am häufigsten genannt wurden Inhalte zur elektronischen Patientenakte (ePA), zum eRezept sowie zu Kommunikationsplattformen wie Kommunikation im Gesundheitswesen (KIM) und Messenger der Telematik (TIM).

Das subjektiv eingeschätzte eHealth-Wissen korrelierte stark mit dem empfundenen Vertrauen im Umgang mit eHealth-Anwendungen sowie mit dem Gefühl der Vorbereitung auf den beruflichen Einsatz. Die wahrgenommene Sicherheit war der stärkste Prädiktor für das Gefühl beruflicher Vorbereitung. Auch Geschlecht und Alter zeigten Zusammenhänge: Männer schätzten ihr Wissen höher ein, jüngere Teilnehmende berichteten häufiger von eHealth-Inhalten im Studium.

**Schlussfolgerung:**

Die Ergebnisse verdeutlichen große Unterschiede in der Wahrnehmung der eHealth-Lehre und zeigen signifikante Zusammenhänge zwischen Wissen, Sicherheit und Vorbereitung auf die digitale Berufspraxis. Die Teilnehmenden äußerten einen deutlichen Bedarf an umfassenderer und strukturierterer Lehre zu digitalen Gesundheitskompetenzen. Zentrale Themen für die zukünftige curriculare Integration sind digitale Dokumentationssysteme, Telemedizin, Datenschutz und der Einsatz von Künstlicher Intelligenz in der Medizin. Eine systematische und langfristige Einbindung dieser Themen in Pflicht- und Wahllehrveranstaltungen ist essenziell, um angehende Ärzt*innen angemessen auf ein zunehmend digitalisiertes Gesundheitssystem vorzubereiten.

## Introduction

The integration of eHealth into medical education varies internationally. For instance, in the United States, eHealth has been incorporated into medical school curricula to prepare students for the digital transformation of the healthcare system [1]. There is evidence that the implementation of eHealth competencies in medical education increases students’ willingness to adopt technological solutions in their future practice [2]. In the Netherlands, a nationwide program has been developed to integrate eHealth into medical education. This program aims to enhance medical students’ digital skills and improve their attitudes toward eHealth. Preliminary findings suggest that such initiatives can foster students’ confidence in using digital technologies [2]. In Australia, Universities have started incorporating eHealth components into their curricula to ensure that future physicians can effectively manage digital health technologies [1], [3].

With the introduction of the Digital Act (DigiG) in 2023, the digitalization of the healthcare sector is set to accelerate in Germany. Among other measures, the electronic prescription (ePrescription) and the electronic patient record (ePA) have been implemented nationwide [4]. However, to practice successfully within these new healthcare structures, appropriate knowledge is required [5].

Research conducted in Germany highlights that a more comprehensive integration of eHealth into medical education is lacking [5], [6], [7]. A significant proportion of medical students express a demand for expanded curricular content in this area to ensure adequate preparation for their professional careers [8]. Similarly, alumni report a continued need for further training in digital health competencies [9]. Recognizing these challenges, the German Medical Association proposed a resolution at the 122^nd^ German Medical Assembly in 2019, emphasizing the urgent necessity of curriculum adjustments to better equip medical students for the increasing digitalization of healthcare [10]. To develop targeted improvements, an initial assessment of the currently taught eHealth-related topics and their scope within medical curricula is essential.

Since 2021, the National Catalogue of Competence-based Learning Objectives for Medicine (NKLM) has been updated to include telemedicine content as a mandatory component. Among other things, students should be able to explain ‘application scenarios for telemedicine and their framework conditions’ as well as ‘telemedicine aspects of emergency medicine’ after completing their studies [11]. Universities in Germany are responding differently to the new digital requirements. For instance, the University of Giessen has developed a specialized curriculum entitled ‘Digital Medicine, eHealth and Telemedicine’ [12]. At the University of Marburg, medical students have the option of taking the elective course ‘Medicine in the Digital Age’ [13].

At the University of Luebeck, eHealth content is currently integrated into the compulsory curriculum, including general medicine (with an OSCE station), medical sociology, and medical informatics [8], [14]. Since 2022, eHealth content has been taught, e.g. in a 90-minute seminar, in the general medicine module [15], [16]. In medical sociology, eHealth content has been taught for six hours since 2019 [8], [15]. The Institute of Family Medicine teaching is basically based on the content of the book ‘Telemedicine and eHealth’, which was published in 2021 [17]. The medical informatics module includes the lecture series ‘Medical Documentation’ and the seminar ‘Health Telematics and Telemedicine,’ which have been taught since 2003 [15], [18]. There is currently no independent module as a compulsory or elective subject on eHealth or telemedicine at the University of Luebeck [14].

The aim of this study was to assess the memory regarding teaching content related to eHealth for medical students for all the subjects at the University of Luebeck. The students’ perception of the eHealth content taught is to be examined. 

## Methods

### Study design and population

A cross-sectional study was conducted using an online questionnaire to assess the integration of eHealth content into the medical curriculum at the University of Luebeck. The study population comprised currently enrolled medical students as well as alumni who graduated within the past five years. At the time of the survey, approximately 1,600 students were enrolled in the medical program at the University of Luebeck [19]. Participants were recruited via email distribution lists from November 2024 until July 2025, ensuring broad coverage of both student and alumni groups. Eligibility criteria included a minimum age of 18 years, proficiency in the German language, and prior written informed consent for study participation. Only individuals meeting these criteria were included in the study sample.

### Ethics and data security

Data collection was conducted for the purpose of the aforementioned research project. The data was recorded, stored, and analyzed in anonymized form. The provision of the General Data Protection Regulation (GDPR) was strictly adhered to [20]. Access to the questionnaire data is restricted to study staff members, all of whom are bound by confidentiality agreements. The data is protected against unauthorized access, and third parties will not have access to the original records. The data was stored at the Institute of Family Medicine, University of Luebeck, and will be deleted after a period of 10 years. The study was approved by by the Ethics Committee of the University of Luebeck on 30.09.2024 (file number: 2024-533).

### Questionnaire

The survey was conducted using the online platform SurveyMonkey (San Mateo, CA) [21]. The data was collected anonymously, ensuring that no conclusions could be drawn about individual participants. The questionnaire contains questions on personal attitudes and usage behavior of eHealth, the current status of teaching units in medical curricula, how confident the participants feel in using eHealth applications and which topics they would have liked to have covered. Demographic information included gender, age, and either the current semester of study or the date of graduation. The questionnaire can be found in Attachment 1.

### Analyses

Data analysis was carried out using IBM SPSS Statistics software (Armonk, NY), version 29 [22]. The data was first analyzed descriptively. Ordinal data were recorded using a 6-point Likert scale. This was based on the school grading system in Germany. 1 stands for very good or very high and 6 for very poor or very low.

In addition, the variables were tested for normal distribution using the Kolmogorov-Smirnov test. If the data was not normally distributed, non-parametric tests were calculated. The Mann-Whitney U test was used for the group comparison. To explore the associations between key variables related to eHealth, non-parametric correlation analyses were conducted using Spearman’s rank-order correlation (Spearman-Rho). Correlations were computed between variables such as general attitude toward eHealth, self-assessed knowledge, perceived confidence in using eHealth applications, and perceived preparedness for professional use. In addition, associations with sociodemographic variables (e.g., gender, age, role) and reported curricular exposure to eHealth were examined. Statistical significance was set at p<0.05 for all analyses. 

## Results

In total, 131 questionnaires were completed. Table 1 (Tab. 1) shows the sociodemographic data of the participants.

Table 2 (Tab. 2) shows the general attitude of the participants towards eHealth and their knowledge of eHealth. It also shows how comfortable and prepared the participants feel. 

Table 3 (Tab. 3) provides an overview of which applications the participants use privately. It also shows whether and which eHealth content was taught in the curriculum.

eHealth was mainly remembered getting taught in the subjects of general medicine (29%), medical sociology (32%) and medical informatics (7%). Individual participants also stated lectures in pharmacology, psychology and prevention and health. 

The respondents were also asked which subjects they would have liked to hear (more of) in the curriculum. The following topics were listed: general overview (“everything”), importance for patient care, data protection, possible applications, legal framework, ePA, ePrescription, Digital health applications (DiGA), video consultation, artificial intelligence (AI), KIM, TIM and messaging apps. 

### Correlational analyses

*Attitude toward eHealth* showed a moderate positive correlation with *knowledge about eHealth* (r_rho_=0.29, p<.001). *Knowledge about eHealth* was strongly positively correlated with *confidence in using eHealth applications* (r_rho_=0.551, p<.001) and *perceived preparedness* (r_rho_=0.514, p<.001). *Confidence in using eHealth applications* was strongly associated with *perceived preparedness* (r_rho_=0.721, p<.001). A small but significant correlation was found between *gender* and *knowledge about eHealth* (r_rho_=–0.240, p=.006), with male participants rating their knowledge slightly higher. The presence of *eHealth content in the curriculum* was positively associated with *perceived preparedness* (r_rho_=0.302, p<.001). *Age* showed a negative correlation with reported *eHealth content in the curriculum* (r_rho_=–0.362, p<.001), suggesting that younger participants more frequently encountered eHealth-related teaching.

### Subgroup analysis

Subgroup analyses were performed with the variables on attitude and knowledge. Table 4 (Tab. 4) shows the group analysis based on the variable on *eHealth content in the curriculum*, showing a difference in perceived preparedness.

The comparison of the subgroups by *Role (students vs. alumni)* with the variables on attitude and knowledge showed that there was no difference in the variables. Table 5 (Tab. 5) shows a difference in *knowledge about eHealth* and *confidence in using eHealth applications*. 

## Discussion

The study sought to determine the scope and specific topics covered in the curriculum at the University of Luebeck. It also aimed to show how comfortable and prepared the participants feel for everyday practice.

Overall, the analyses support the assumption that *knowledge about eHealth*, *confidence in using eHealth applications*, and *eHealth content in curriculum* are mutually reinforcing factors and are linked to a more positive *attitude toward eHealth* and *perceived preparedness*. Correlational analyses within our study emphasize the critical relationship between self-assessed eHealth knowledge and students’ readiness and confidence in applying eHealth professionally. These findings are consistent with other studies, who noted that students often feel underprepared for digital practice but express a strong desire for more robust training [2], [23], [24]. Our data revealed that the confidence in using eHealth applications and the perceived preparedness for the job are strongly correlated, echoing prior work that suggested experiential learning significantly boosts digital competence [25], [26]. The taught eHealth content in the curriculum and its influence on the perceived preparedness for the job highlight the layered nature of digital competency, suggesting that fostering confidence through hands-on, clinically relevant training is essential. This is corroborated by the success of structured, multi-institutional telemedicine curricula which yielded high student satisfaction and knowledge gain [25], [27].

The role of gender also warrants attention, as male students rated their *knowledge about eHealth* higher than the female participants did, consistent with previously documented gender differences in digital self-efficacy [28]. This aligns with other research. It is consistently indicated that men tend to overestimate their abilities, while women often underestimate theirs, despite comparable actual performance levels. This phenomenon, sometimes referred to as the “male hubris, female humility” effect, has been observed across various domains [29], [30]. These disparities in self-assessment have tangible implications. A study examining medical students’ self-evaluations found that female students rated their performance significantly lower than peer assessments, while male students’ self-assessments closely matched peer evaluations. Such underestimation by women can influence career advancement opportunities, as self-promotion plays a crucial role in professional growth. Understanding and addressing these gender-based differences in self-perception is essential for fostering equity in educational and professional settings [31].

The age of the participants and the Content of eHealth in Curriculum were inversely correlated, indicating that younger students may benefit from newer curriculum reforms or increased digital exposure during early training.

Students’ demand for broader eHealth content – ranging from data protection and AI to ePrescriptions and ePA – furtherr underscores a curricular gap. This reflects findings from other studies, who reported a lack of systematic coverage of emerging technologies like AI and Internet of Things (IoT) despite their growing relevance in clinical practice [3]. The integration of eHealth education into medical curricula remains inconsistent, as reflected in our findings. This aligns with earlier research indicating a fragmented approach to digital health training, often relegated to elective modules or isolated teaching units without standardized curricula across institutions [1], [8]. Notably, the preference for general medicine and sociology as instructional contexts may stem from their adaptability to evolving topics such as patient communication, digital literacy, and systems thinking. This trend is evident in German programs, where medical sociology has been a common platform for exploring digital transformation themes [8]. Both medical sociology and general medicine are proposed in the NKLM in order to include telemedicine content in the curriculum [11]. The minimal inclusion in medical informatics is concerning, particularly given that most digital tools and applications are inherently rooted in informatics principles [32]. However, it should be considered that it is also possible that students may not remember the eHealth aspects that were taught. In this case, a new implementation strategy could help to sustainably promote knowledge among students. For instance, it would be conceivable to include an additional elective subject on eHealth in the curriculum. This has already been successful at the University of Mainz and could also be adapted in Luebeck [33], [34]. The evaluation showed that hands-on components and practical content were particularly conducive to learning success. The methodological diversity and scope of the modules were also highlighted as positive aspects [26]. In terms of methodology, particular attention should be paid to ensuring that students are actively involved and that what they have learned is repeated promptly [26], [35]. Digital health education should be systematically embedded in both core and elective curricula to ensure that all students – not just those opting into specialized modules – are equipped for digital practice [27], [32]. Increasing the number of longitudinal planned teaching units on the subject of eHealth may be useful in order to ensure that the topics are remembered in the long term. In doing so, the university should create the conditions for incorporating new content by reducing other to not overstrain the students and enable teaching staff to catch up with the developments in this field [24].

One aspect that was not considered in this study is the eLearning platform for students provided by the University of Luebeck. It is available to all students and here as well, eHealth content is available for further research [36]. Whether and to what extent this is used by the students is not shown in this study. This should be taken into account in the further development of eHealth content in the curriculum.

### Strengths and limitations

A strength of this study is that all current students (1^st^ of November 2024) from the University of Luebeck were contacted via email distribution lists. Nevertheless, response rates cannot be calculated as different university mailing lists were used. Students and alumni may appear on more than one mailing list and may have received the request twice or never. It is not possible to draw conclusions about how many alumni have been contacted, as there is no number available. Due to the small number of participants, the results cannot be extrapolated to the entire student body at the University of Luebeck. Despite anonymous questionnaires, it is possible that the participants were biased towards answers they perceived to be desirable. In addition, recall bias is possible and must be considered in the interpretation, especially among alumni, since their studies were completed a few years ago. It should also be noted that students in their first to third semesters could not yet have covered eHealth content, as this is only taught for the first time in the fourth semester in Medical Sociology. It is also important to consider that people who are interested in the topic of telemedicine are more inclined to take part in a survey [37], [38].

## Conclusion

This study highlights the current limitations and potential of eHealth education within medical curricula. While subjects such as general medicine and medical sociology provide an entry point for eHealth-related topics, the low representation in other subjects and the low number of lessons emphasizes a significant structural gap. It is also shown, that despite the fact that eHealth is taught, it is not remembered much by students. Given the strong associations between self-assessed eHealth knowledge, confidence, and perceived professional preparedness, it is imperative that digital health training be expanded and better integrated into core curricula.

Based on the results of the study, aspects such as the fundamentals and implications of digital health technologies, data protection and legal framework and secure communication platforms such as KIM and TIM should be (more) integrated into the curriculum. This would meet the needs expressed by students from the University of Luebeck and the demands of an increasingly digital healthcare system.

Incorporating these components into both compulsory teaching formats is essential not only to prepare future physicians for everyday practice but also to foster a more positive, confident, and competent engagement with digital health innovations. Without a standardized and comprehensive approach, the potential of eHealth in improving patient care and clinical efficiency will remain underutilized.

## Notes

### Funding

This research was supported by resources provided by the Institute of Family Medicine of the University Medical Centre Schleswig-Holstein, Campus Luebeck.

### Competing interests

JS received public funding for several eHealth projects and he is the editor of a book on eHealth and telemedicine. The other authors have no competing interests as defined by GMS, or other interests that might be perceived to influence the results and/or discussion reported in this paper.

## Supplementary Material

Questionnaire

## Figures and Tables

**Table 1:**
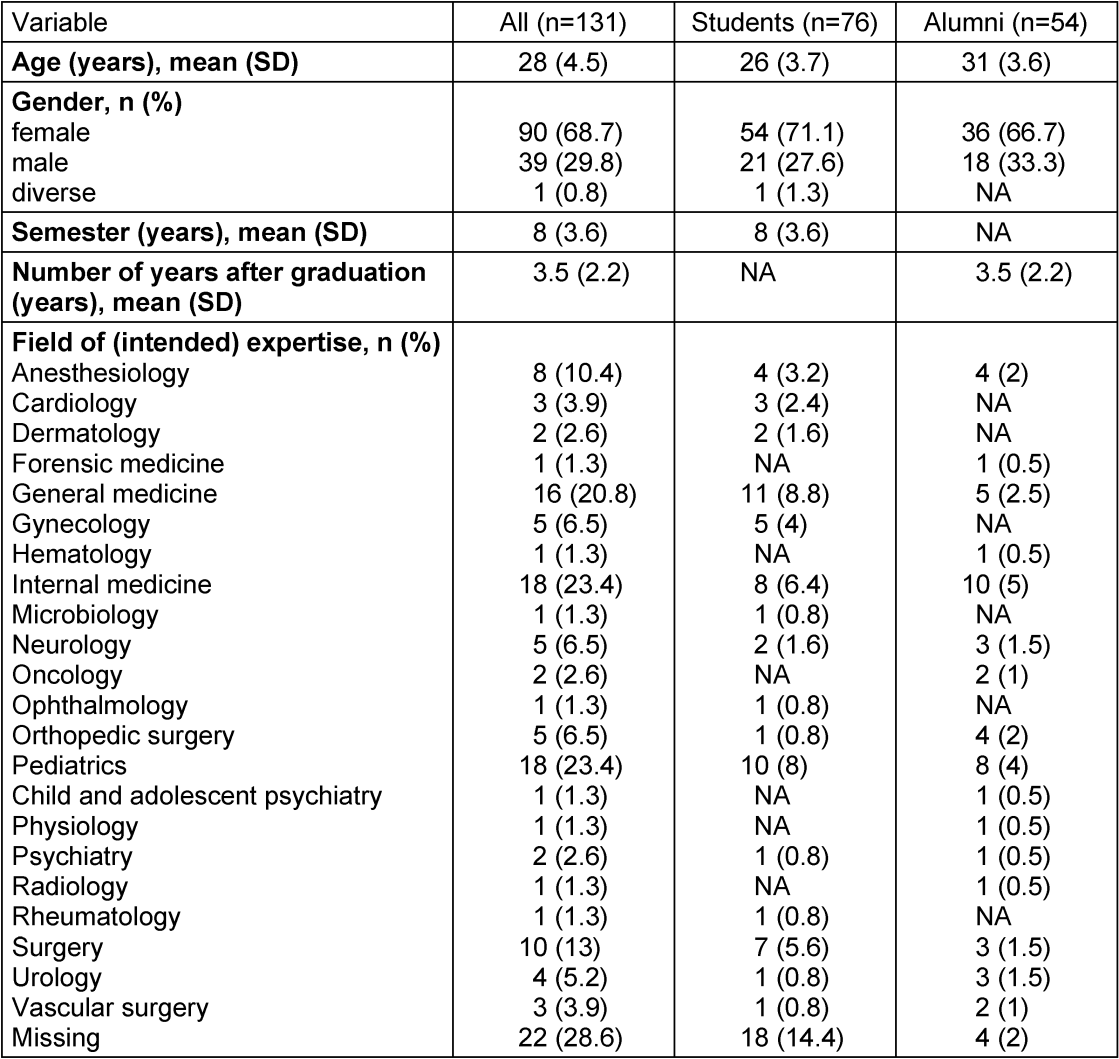
Sociodemographic of the participants

**Table 2:**
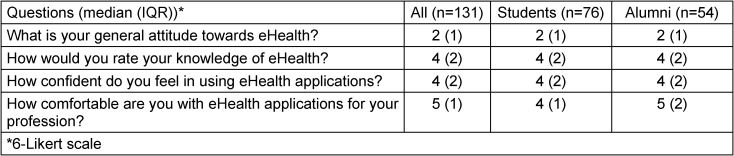
Attitudes and knowledge about eHealth

**Table 3:**
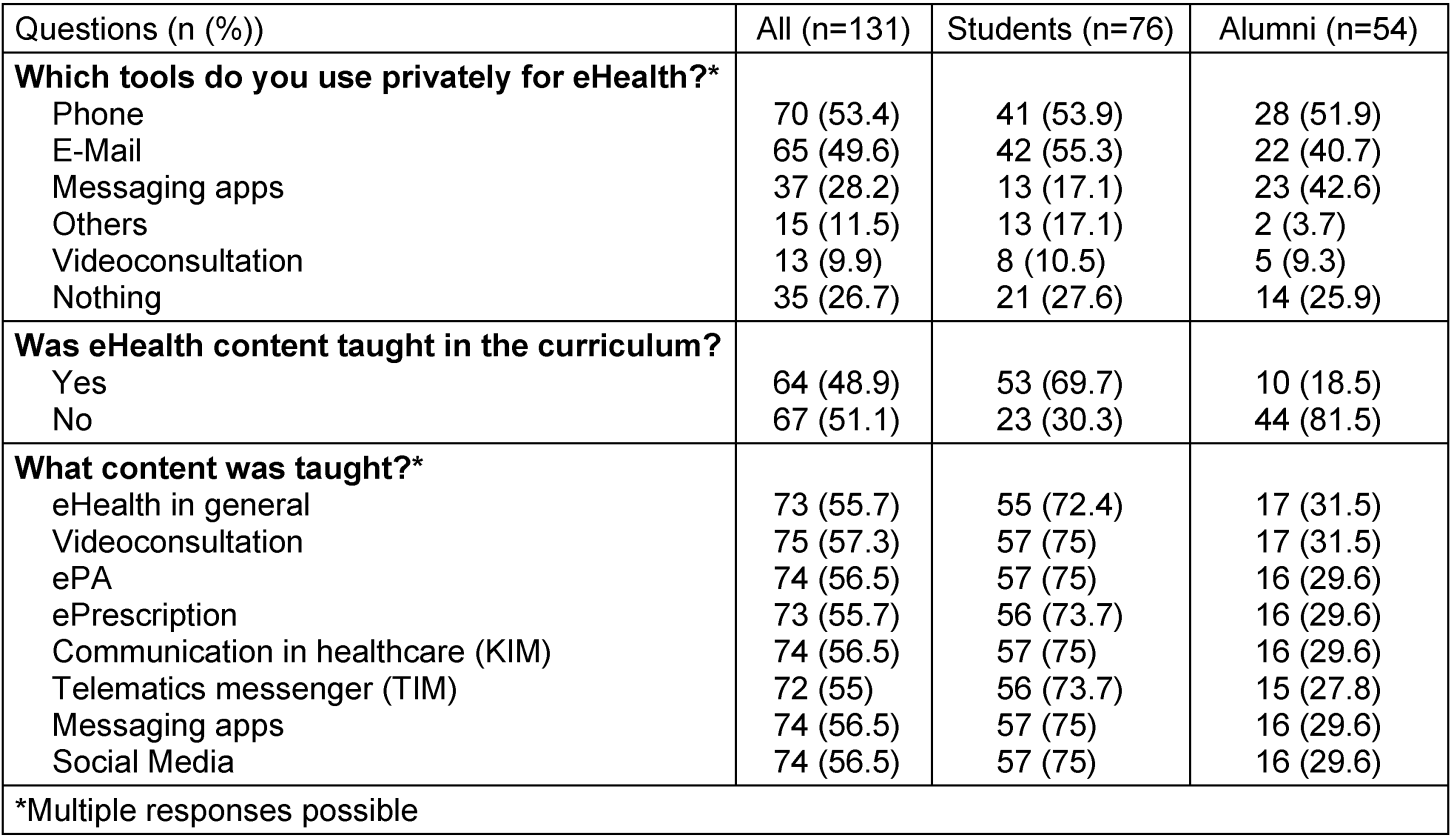
User behavior and academic content about eHealth

**Table 4:**
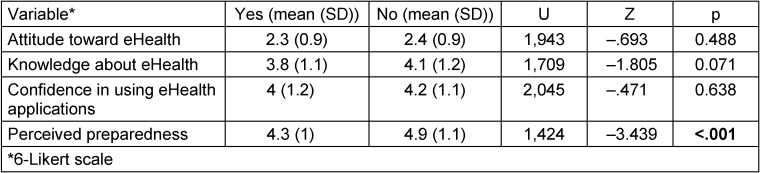
Attitude and knowledge concerning the availability of eHealth content in the curriculum (yes vs. no): a group comparison using Mann-Whitney-U test

**Table 5:**
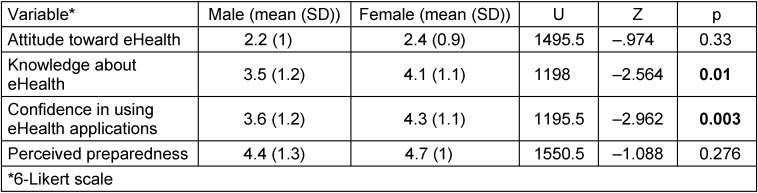
Comparison of the subgroups by gender (male vs female) using Mann-Whitney-U test
