# Supplementary material for: Requirements and attitudes toward eHealth content in human medicine studies: A cross-sectional study to improve the curriculum
Source: Ger Med Sci. 2026 Jan 13;24:Doc02. doi: 10.3205/000355 (PMC12914370; doi:10.3205/000355)
Supplement: Questionnaire [file GMS-24-02-s-001.pdf]

UNIVERSITÄT ZU LÜBECK
INSTITUT FÜR ALLGEMEINMEDIZIN

eHealth in human medicine studies at the University of Lübeck

Dear students and alumni of the University of Lübeck,

The Institute of Family Medicine at the University Hospital Schleswig-Holstein on the Lübeck campus is conducting a survey on the topic of eHealth in teaching at the University of Lübeck.

Your responses will help us to gain and identify insights and areas for improvement in teaching on the subject of eHealth.

The questionnaire will take about 10 minutes to complete.

If you have any questions about this project, please contact:

Contact person: Pia Traulsen

Phone number: +49 451 31018021

Email: pia.traulsen@uni-luebeck.de

Instructions for completing the questionnaire

By submitting your questionnaire, you consent to the Institute of General Medicine evaluating and publishing your information. As the questions are collected anonymously, you cannot withdraw from the study once you have submitted the questionnaire.

Privacy policy:

Data is collected for the purposes of the above-mentioned research project. Your data will be stored and evaluated electronically in anonymous form. The provisions of the General Data Protection Regulation (GDPR) are complied with. Only employees involved in the study have access to the data contained in the questionnaires. These individuals are bound to confidentiality. The data is protected against unauthorized access. Third parties do not have access to the original documents.

Thank you for your support!

Attachment 1 to: *Traulsen P, Stamer T, Steinhäuser J. Requirements and attitudes toward eHealth content in human medicine studies: A cross-sectional study to improve the curriculum. GMS Ger Med Sci. 2026;24:Doc02.*
DOI: 10.3205/000355, URN: urn:nbn:de:0183-0003559

	Very positive					very negative
**1.** What is your general opinion towards eHealth?	❑	❑	❑	❑	❑	❑

	very high					very low
**2.** How would you rate your knowledge of eHealth?	❑	❑	❑	❑	❑	❑

**3.** Which tools do you use privately for eHealth?	
Phone	❑
Messaging-App (z. B. WhatsApp, Siilo)	❑
E-Mail	❑
Videoconsultation (z. B. Arztkonsultation, Patientus)	❑
Others: ______________________	❑
Nothing	❑

4. Was eHealth content taught in the curriculum? ❑ yes ❑ no (continue with question 7)

**5.** What content was taught?	Yes	No
eHealth in general	❑	❑
Videoconsultation	❑	❑
ePA (electronic patient record)	❑	❑
ePrescription	❑	❑
Communication in healthcare (KIM)	❑	❑
Telematics messenger (TIM)	❑	❑
Messaging-App (z. B. Siilo)	❑	❑
Social Media	❑	❑
Others: ______________________	❑	❑

Attachment 1 to: *Traulsen P, Stamer T, Steinhäuser J. Requirements and attitudes toward eHealth content in human medicine studies: A cross-sectional study to improve the curriculum. GMS Ger Med Sci. 2026;24:Doc02.*
DOI: 10.3205/000355, URN: urn:nbn:de:0183-0003559

**6.** In which modules were eHealth topics covered during your studies?
❑ general medicine<br>❑ medical sociology<br>❑ medical information<br>❑ others: __________________

	Very confident					very insecure
**7.** How confident do you feel in using eHealth applications?	❑	❑	❑	❑	❑	❑

	Very comfortable					very uncomfortable
**8.** How comfortable are you with eHealth applications for your profession?	❑	❑	❑	❑	❑	❑

**9.** What content related to eHealth would you have liked to learn (more) about during your studies?

Attachment 1 to: *Traulsen P, Stamer T, Steinhäuser J. Requirements and attitudes toward eHealth content in human medicine studies: A cross-sectional study to improve the curriculum. GMS Ger Med Sci. 2026;24:Doc02.*
DOI: 10.3205/000355, URN: urn:nbn:de:0183-0003559

10. Personal information:

a) Your gender? ❑ male ❑ female ❑ diverse

b) What is your year of birth? ☐ ☐ ☐ ☐

c) Please tick the appropriate box: I am a(n) ❑ student ❑ alumni

d) For students: Which semester are you? ☐ ☐

e) Which field are you (interested in) pursuing?

f) For alumni: In what year did you graduate? ☐ ☐ ☐ ☐

**Comments:**

Attachment 1 to: *Traulsen P, Stamer T, Steinhäuser J. Requirements and attitudes toward eHealth content in human medicine studies: A cross-sectional study to improve the curriculum. GMS Ger Med Sci. 2026;24:Doc02.*
DOI: 10.3205/000355, URN: urn:nbn:de:0183-0003559
